# Co-Twin Prognosis After Single Intrauterine Fetal Death at a Tertiary Care Hospital in India: A Retrospective Observational Study

**DOI:** 10.7759/cureus.63336

**Published:** 2024-06-27

**Authors:** Tanvi Katoch, Snigdha Kumari, Anju Singh, Vanita Suri, Rashmi Bagga, Jogender Kumar

**Affiliations:** 1 Obstetrics and Gynaecology, Postgraduate Institute of Medical Education and Research, Chandigarh, IND; 2 Pediatric Medicine, Postgraduate Institute of Medical Education and Research, Chandigarh, IND

**Keywords:** neonatal death, monochorionic twins, dichorionic twins, preterm birth, twin pregnancy

## Abstract

Introduction: Twin pregnancy is associated with an increased risk of perinatal morbidity. Besides, if intrauterine death of a single twin occurs, it increases the morbidity of the surviving co-twin perinatally and postnatally.

Aim: The objective of this study was to determine the incidence of single intrauterine fetal death (SIUFD) in a twin pregnancy and fetal outcome defined in dimensions according to the complications in the surviving co-twin.

Material and methods: Data on twin pregnancies were collected retrospectively for a period of five years (from 2015 to 2019) from the labour room records of the Central Records Department (CRD) at the Postgraduate Institute of Medical Education and Research, Chandigarh, India. Cases with SIUFD were studied individually and neonatal follow-up was taken post delivery for up to three to eight years. Inclusion criteria were SIUFD in twin pregnancies after 14 weeks gestation, chorionicity pre-defined by early trimester ultrasonography. Exclusion criteria were higher-order pregnancy and monoamniotic twins.

Results: A total of 1246 (4.273%) twin deliveries were conducted in the study period. Of these, 107 (8.587%) pregnancies had SIUFD with co-twin surviving in utero. Among these, 77 (72%) were dichorionic diamniotic (DCDA) twin pregnancies and 30 (28%) were monochorionic diamniotic (MCDA) twin pregnancies. The incidence of SIUFD was 8.5%. Preterm birth was the most common complication observed in our study and was found in 53.5% and 58.3% of participants in DCDA and MCDA twins, respectively. Early neonatal death (within 24 hours of life) of the surviving twin was found in 29.2% monochorionic twins with SIUFD. SIUFD at < 28 weeks gestation led to a greater number of early neonatal deaths of surviving twins. The incidence of neurodevelopmental disorders (cerebral palsy, developmental delay, epilepsy) in our population after birth was 7.5% (n=93).

Conclusion: Twin pregnancies with SIUFD have an increased incidence of preterm labour, increased neonatal death of the surviving twin, and neurodevelopmental disorders (cerebral palsy, developmental delay, epilepsy). Monochorionicity and SIUFD at <28 weeks gestation are associated with increased neonatal deaths in co-twin. The Incidence of neurodevelopmental disorders is not directly associated with chorionicity, but developmental delay is more profoundly seen in the monochorionic group.

## Introduction

Twin pregnancy is intriguing as it poses an increased risk of perinatal morbidities and mortality [1,2]. The risk increases in case of intrauterine fetal death of a single twin [1]. This affects the mother and the surviving co-twin, in several manners, some of which have proposed mechanisms [3]. According to the literature, approximately 6% of twin pregnancies are affected by single intrauterine fetal death (SIUFD), though it does not indicate the true prevalence of the same [1-3]. SIUFD diagnosed in a dating scan before 14 weeks is termed as vanishing twin, but SIUFD occurring after 14 weeks in a twin pregnancy has been seen to lead to consequences for the mother as well as surviving co-twin [4]. Fetal effects are the death of the surviving twin, preterm birth, restricted growth and neurological developmental abnormalities due to brain injury [5-7]. The morbidity is more in surviving co-twins in monochorionic twins, as is the incidence, likely due to placental intertwin anastomoses [5]. 

Due to the anticipation of the above complications, and uncertain incidence of same, the clinical management, surveillance, and timing of termination of twin pregnancy with SIUFD are variable and remain controversial [8]. The objective of this study was to determine the incidence of SIUFD in a twin pregnancy and fetal outcome defined in dimensions according to the complications of the same, that presented in a tertiary Indian setup.

## Materials and methods

This was a retrospective study conducted at the Postgraduate Institute of Medical Education and Research (PGIMR), Chandigarh, India. The study was approved by the Institutional Ethics Committee, PGIMR (approval number: INT/IEC/2021/SPL-1085). Data on twin pregnancies were collected retrospectively for a period of five years (from January 2015 to December 2019) from the Labor Room records in the Central Records Department (CRD) of PGIMR. Cases with SIUFD were studied individually and neonatal follow-up was available post delivery ranging from three to eight years. Inclusion criteria were SIUFD in twin pregnancy after 14 weeks gestation, chorionicity pre-defined by early trimester ultrasonography. Exclusion criteria were higher-order pregnancy and monoamniotic twins.

Demographic details and obstetric history of the study population were noted. The chorionicity of the twins was assessed by first-trimester ultrasound. Any preexisting or coexisting fetal complications such as discordant structural anomaly, discordant growth, twin-to-twin transfusion syndrome, and placental insufficiency were noted. Similarly, maternal complications such as hypertension, diabetes mellitus or gestational diabetes, cholestasis of pregnancy, thyroid disorder, or other medical comorbidities were also noted. The gestational age at which SIUFD occurred was noted. Any complications or events occurring after SIUFD such as selective fetal growth restriction (FGR), placental insufficiency, preterm rupture of membranes and prelabour rupture of membranes, placental abruption, symptomatic placenta previa (antepartum haemorrhage), preterm labour, and hypertensive emergencies were also noted.

Delivery of the co-twins is dependent on maternal reasons or if non-reassuring signs of fetal well-being of the co-twins were found at ultrasound scans or non-stress tests, suggesting early delivery. Chorionicity, gestational age, and mode of delivery were noted. The outcomes of the surviving twin were assessed and birthweight, APGAR score, and early or late neonatal complications were noted. The surviving twins were followed for neurological outcomes (cerebral palsy, developmental delay, or epilepsy) or any other comorbidity for three to eight years. The study aimed to determine the incidence of SIUFD in twin pregnancies and the aetiology of SIUFD in twin pregnancies. The overall prognosis of the surviving twin was assessed by growth restriction in the surviving twin, timing of birth, neonatal complications and neonatal death, and neurological abnormalities after birth.

Statistical analysis

The data was entered and analysed in IBM SPSS Statistics for Windows, Version 24.0 (Released 2016; IBM Corp., Armonk, NewYork, United States). The data was checked for its normality and skewness using Kolmogorov-Smirnov test. The univariate analysis was done and represented by frequency, percentages, mean, standard deviation, median, and interquartile intervals. Bar charts were used to present categorical data. The chi-square test was used to find the association between dependent variable and independent variables. The odds ratio was calculated to find the strength of the association between dependent and independent variables. p-values <0.05 were taken as statistically significant with a 95% confidence interval. Continuous data was categorized into three groups for representing the distribution of data; however, the gestation age at intrauterine fetal death was categorised into two groups (taking less than and more than 28 weeks) for analysis.

## Results

Over five years, 29,159 deliveries took place at our centre, of which 1246 (4.273%) were twin deliveries. Of all twin deliveries, 107 (8.587%) pregnancies had SIUFD with the co-twin surviving in utero. Among these, 77 (72%) were dichorionic diamniotic (DCDA) twin pregnancies and 30 (28%) were monochorionic diamniotic (MCDA) twin pregnancies. The demographic details and antenatal and intrapartum characteristics of the study population are listed in Table 1. The majority of the pregnant women were of average reproductive age (mean age was 30.1 ± 4.8 years). Ninety-six (89.7%) of all had a preterm birth (<37 weeks of gestation) of which 11 were iatrogenic. Of the 107 SIUFD cases, 58 (54.2%) newborns had a birth weight <1500 grams, 41 (38.3%) had a weight of 1500-2500 grams, and eight (7.5%) had a birthweight >2500 grams.

**Table 1:** Demographic details and antenatal and intrapartum characteristics of participants (N=107)

Characteristics		Number	Percentage
Age	21-25 years	17	15.9
	26-35 years	78	72.9
	36-47 years	12	11.2
Gravidity	Multigravida	49	45.8
	Primigravida	58	54.2
Previous live issues	0	80	74.8
	1	20	18.7
	2	6	5.6
	3	1	0.9
Previous Abortions	0	85	79.4
	1	17	15.9
	2	3	2.8
	3	1	0.9
	4	1	0.9
Previous stillbirths	0	97	90.7
	1	8	7.5
	2	1	0.9
	3	1	0.9
Nature of conception	In vitro fertilisation	14	13.1
	Natural	87	81.3
	Ovulation induction	6	5.6
Gestation at delivery	<37 weeks	96	89.7
	> 37 weeks	11	10.3
Delivery details	Spontaneous onset of labour	58	54.2
	Induction of labour	11	10.3
	Vaginal delivery	54	50.5
	Caesarean delivery	53	49.5
Birthweight	<1500 grams	58	54.2
	1501-2499 grams	41	38.3
	>2500 grams	8	7.5

**Figure 1:**
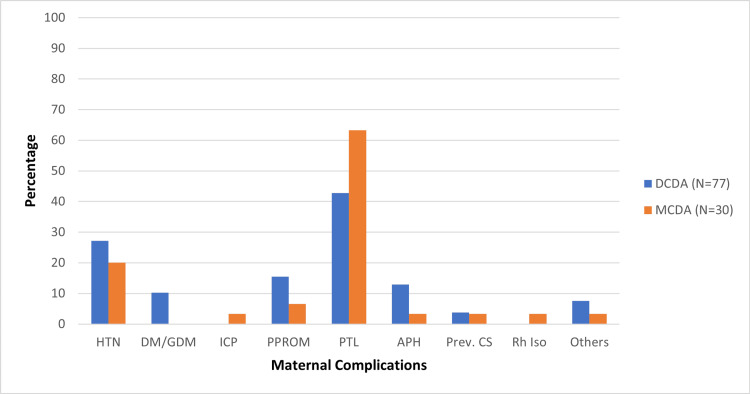
Distribution of maternal complications among study participants DCDA: dichorionic diamniotic twins; MCDA: monochorionic diamniotic twins; HTN: hypertension; DM/GDM: diabetes mellitus/ gestational diabetes mellitus; ICP: intrahepatic cholestasis of pregnancy; PPROM: preterm prelabour rupture of membranes; PTL: preterm labour; APH: antepartum haemorrhage; Prev. CS: previous caesarean section; Rh Iso: Rh isoimmunisation

Eighty-nine (83.2%) women had one or more maternal complications during pregnancy and 64 women (59.8%) had fetal complications. The maternal complications and their occurrence among DCDA and MCDA twins are depicted in Figure 1. Preterm labour was the commonest complication in both DCDA and MCDA groups. Among all, 52 (48.5%) women, with 33 (42.8%) in the DCDA group and 19 (63.3%) in the MCDA group, went into preterm labour.

**Figure 2:**
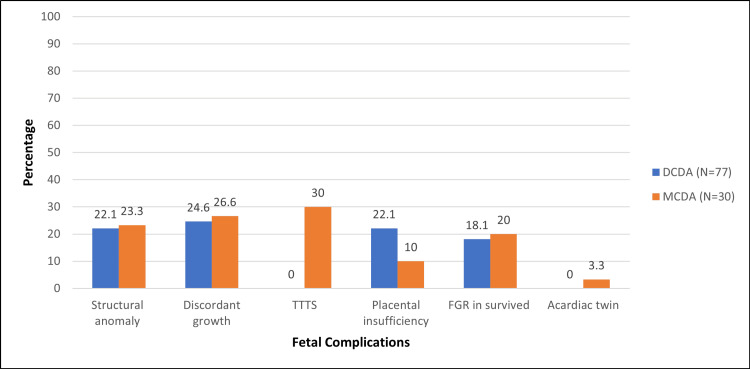
Distribution of existing fetal complications among study participants DCDA: dichorionic diamniotic twins; MCDA: monochorionic diamniotic twins; TTTS: twin-to-twin transfusion syndrome; FGR: fetal growth restriction

The existing fetal complications in both groups are depicted in Figure 2. These complications occurred almost equally common in DCDA (N=77) and MCDA (N=30) twins, except for placental insufficiency. A total of 20 had placental insufficiency of which 17 were DCDA twins (22.1% of total DCDA) and three were MCDA twins (10% of total MCDA) (OR= 2.55, p<0.05). Besides, in the MCDA twins, nine (30%) had twin-to-twin transfusion syndrome (three underwent radiofrequency ablation) and one (3.3%) had an acardiac twin.

**Figure 3:**
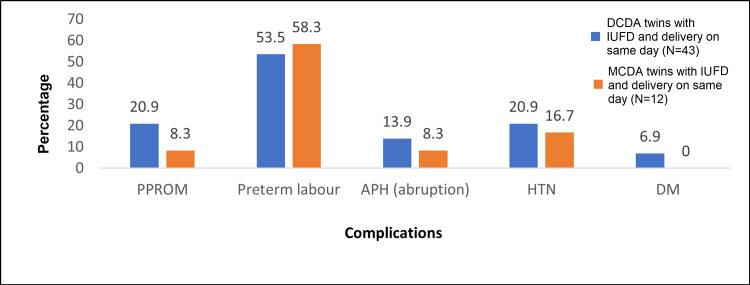
Distribution of complications among participants having SIUFD and delivery on same day. DCDA: dichorionic diamniotic; MCDA: monochorionic diamniotic; PPROM: preterm prelabour rupture of membranes; APH (abruptio): abruption-related antepartum haemorrhage; HTN: hypertension; DM: diabetes mellitus; IUFD: intrauterine fetal demise

Forty-three (55.8%) DCDA and 12 (40%) MCDA twins were delivered on the day of the IUFD, suggesting an association between intrauterine fetal death and the nature or cause of delivery. Complications among women having SIUFD and delivery on the same day are shown in Figure 3. 

The mean gestation age at IUFD was 28.7 ± 4.9 weeks and the mean gestation of delivery was 31.9 ± 3.4 weeks. The median birthweight of the surviving twin was 1368 grams. Of 107 candidates, only 93 could be followed further for post-natal events. Out of 93 survived live-born co-twins, 69 were DCDA and 24 were MCDA. The complications in the neonatal and infantile period of the surviving twins are enumerated in Table 2.

**Table 2:** Distribution of neonatal complications and their occurrence based on chorionicity (N=93) DCDA: dichorionic diamniotic twins; MCDA: monochorionic diamniotic twins; CI: confidence interval; SGA: small for gestational age. The cells denoted with single hyphen "-" are the ones in which OR and 95%CI could not be calculated as the respective complication had occurred in none of the neonates/mothers in either of the two groups (DCDA and MCDA).

Characteristics	DCDA (n=69), n (%)	MCDA (n=24), n (%)	Total (N=93), n (%)	Odds Ratio	95% CI	p-value
No neonatal complication	13 (18.8)	4 (16.6)	17 (18.2)	1.16	0.33-3.97	0.81
Respiratory distress	36 (52.2)	16 (66.7)	52 (55.9)	0.54	0.20-1.44	0.21
SGA	13 (18.8)	6 (25)	19 (20.4)	0.69	0.23-2.10	0.51
Jaundice	17 (24.6)	8 (33.3)	25 (26.9)	0.65	0.23-1.79	0.40
Sepsis	16 (23.2)	6 (25)	22 (23.7)	0.90	0.30-2.66	0.85
Neurodevelopmental disorder	5 (7.2)	2 (8.3)	7 (7.5)	0.85	0.15-4.75	0.86
Cerebral palsy	3 (4.3)	1 (4.2)	4 (4.3)	1.04	0.104-10.55	0.97
Epilepsy	1 (1.4)	0	1 (1.1)	-	-	0.55
Delayed milestones	4 (5.8)	3 (12.5)	7 (7.5)	0.43	0.08-2.08	0.28
Anatomical defects	3 (4.3)	1 (4.2)	4 (4.3)	1.04	0.104-10.55	0.97
Asthma/ rec sinusitis	1 (1.4)	1 (4.2)	2 (2.2)	0.33	0.02-5.62	0.42
Death on day 0	9 (13.0)	7 (29.2)	16 (17.2)	0.36	0.118-1.12	0.07
Death after complications	8 (11.5)	0	8 (8.6)	-	-	0.08
Late infantile death	2 (2.9)	0	2 (2.2)	-	-	0.39
Maternal death	2 (2.8)	0	2 (2.2)	-	-	0.39

Overall, more neonatal complications were found in the MCDA group (83.3%; n=24) when compared with the DCDA group (81.1%; n=69). Respiratory distress was found in 52 (55.9%) of the surviving 93 neonates. Distress, neonatal jaundice, and small for gestational age babies were found more in MCDA twins as compared to DCDA surviving twins. However, chorionicity was found to have no association with the occurrence of early neonatal respiratory distress, small for gestational age babies, neonatal jaundice, or sepsis. A total of 16 (17.2%) neonatal deaths occurred within 24 hours of birth, and a higher percentage of the MCDA neonates died (29.2%, n=24) than DCDA neonates (13%, n=69) (p = 0.07). Neonatal deaths after surviving complications were also more in the MCDA group (p = 0.08)

Monochorionicity was found to be associated with a lesser number of neonates having no complications (OR= 1.16, p=0.8), more neonates with cerebral palsy (OR= 1.04, p=0.97), and more pregnancies having placental insufficiency (OR= 2.55); however, these findings were not statistically significant. Out of 93 survived live-born co-twins, neurodevelopmental disorder occurred in seven (7.5%) and cerebral palsy occurred in four (4.3%), and both entities were found equally likely in both groups. Delayed milestones were found more in MCDA co-twins (three co-twins out of 24, i.e. 12.5%) than in DCDA co-twins (four co-twins out of 69, i.e. 5.8%), but the difference was not statistically significant (OR= 0.43, p=0.28). Late complications that were neurodevelopmental disorder, epilepsy, delayed milestones, anatomical defects, asthma, and late infantile death were found to have no association with chorionicity.

To evaluate the impact of gestation age at SIUFD on the other live twin, the participants were divided into two groups: SIUFD at less than 28 weeks of gestation and SIUFD at more than 28 weeks of gestation. Table 3 shows the distribution of complications occurring in the neonates in whom the other twin had IUFD at less than and more than 28 weeks of gestation. SIUFD at < 28 weeks of gestation was found to result in a greater number of live twins having respiratory distress (OR= 1.24, p=0.61); however, it was not statistically significant. It was also found to result in a greater number of live twins dying on day 0 of life (OR= 5.55, p=0.0048), which was a statistically significant finding. Apart from the above, the gestational age at SIUFD was not found to be associated with small for gestational age, jaundice, sepsis, stay in neonatal intensive care unit (NICU), neurodevelopmental disorder, cerebral palsy, delayed milestones, or death after neonatal complications in the surviving twin.

**Table 3:** Distribution of complications occurring in the neonates in whom the other twin had IUFD at less than and more than 28 weeks of gestation (N= 93) IUFD: intrauterine fetal death; SGA: small for gestational age

Neonatal complications	IUFD at gestational age < 28 weeks (n=39), n	IUFD at gestational age > 28 weeks (n=54), n	Odds Ratio (total)	95% CI	p-value
Respiratory distress	23	29	1.24	0.53 – 2.84	0.6134
SGA	7	12	0.76	0.27 – 2.16	0.6140
Jaundice	8	17	0.56	0.21 – 1.47	0.2391
Sepsis	8	14	0.73	0.27 – 1.98	0.5444
Neurodevelopmental disorder	2	5	0.53	0.09 – 2.88	0.6949
Cerebral palsy	1	3	0.45	0.04 – 4.47	0.6368
Delayed milestones	2	5	0.53	0.09 – 2.88	0.6949
Death on day 0	12	4	5.56	1.63 – 18.9	0.0048
Deaths after complication	3	5	0.81	0.18 – 3.6	1.0000
Late infantile death	0	2	-	-	0.282

## Discussion

Twin pregnancy is associated with more complications as compared to singleton pregnancy [1]. SIUFD is one of its rare complications [1,2]. SIUFD can increase the risk of live co-twin complications during the fetal period, as well as after birth [1,9]. SIUFD occurring during the early embryonic period does not lead to complications in surviving twins [10]. In monochorionic twin pregnancies, there are two mechanisms proposed leading to damage to the surviving co-twin after SIUFD. The older theory is the altered coagulation of the surviving twin due to the passage of thrombi from the dead fetus through anastomotic channels. The recent theory is the reversal of shunt from the co-twin through the anastomotic channels due to hypovolemia in the dead fetus, causing episodes of hypovolemic parenchymal damage, especially neurological, in the surviving twin [3,6,11,12]. In dichorionic twin pregnancies, the live co-twin is compelled to generate an environment for survival besides a dead fetus in the same uterus. There is a lack of a proposed mechanism in the literature on how this affects the surviving twin and the pregnancy [13]. Death of the co-twin, poor neurological outcomes, and preterm birth are seen more frequently in these pregnancies [6]. According to the literature, after SIUFD, the most important factors affecting the prognosis of the live co-twin are gestational age at birth and chorionicity [3,9,14]. Few studies have shown that due to the peculiar angioarchitecture, monochorionic twins are associated with an increased risk of SIUFD and consequential fetal morbidity [2,3,11,14]. The incidence of SIUFD in our study was 8.5%, which is similar to the incidence mentioned in previous studies (0.5-7%) [2,3].

Preterm birth was the most common complication observed in our study, occurring in 49.5% of total study participants (N=107). It was seen in 42.8% of the 77 DCDA pregnancies and 63.3% of the 30 MCDA pregnancies. In a meta-analysis by Mackie et al., preterm birth occurred in 58.5% and 53.7% of monochorionic and dichorionic twin pregnancies [5]. In a similar study by Fichera et al., the median age of delivery in pregnancies with SIUFD was 36 weeks, and 24% delivered before 32 weeks [15]. Their outcome was similar to the study by Ong et al. [6]. Morris et al. performed a study on perinatal outcomes in monochorionic pregnancies with SIUFD and concluded that three-fourths of pregnancies were complicated by preterm birth (delivered at less than 37 weeks) [8].

In our study, early neonatal death (within 24 hours of life) of the surviving twin was found more commonly in monochorionic twins (with single IUFD) (29.2% (n=24) in the MCDA group versus 13% (n=69) in the DCDA group). Mackie et al. found neonatal death in 27.9% MCDA and 21.2% DCDA pregnancies and concluded that there is significantly increased neonatal death of co-twin if SIUFD occurred in monochorionic twins [5]. Similarly, Fichera et al. also found 8.3% neonatal mortality in the MCDA group whereas none in the DCDA group [15]. However, in the study of monochorionic twins with SIUFD by Morris et al., only 3.22% (2/62) neonatal mortality was reported, and they attributed it to extreme prematurity [8]. In our study, SIUFD at < 28 weeks of gestation led to a greater number of early neonatal deaths of surviving twins. This was consistent with the study of Mackie et al. [5].

The incidence of neurodevelopmental disorders in our population after birth was 7.5%. In the study by Fichera et al., neurological results in surviving monochorionic twins were better than the dichorionic group [10]. The neurological sequelae were found in only one surviving dichorionic twin and it was attributed to a suspected perinatal infection. However, Ong et al. concluded a risk of 18% neurological morbidity in the monochorionic group [6]. In the prospective study by Morris et al. which included only a monochorionic group with SIUFD, 7/62 had abnormal postnatal CNS imaging; however, appropriate follow-up was lacking to conclude the clinical manifestations of the findings [7]. In the meta-analysis by Mackie et al., there was a significant difference in the number of surviving twins having abnormal postnatal CNS imaging in monochorionic and dichorionic groups (43% versus 21.2%) [5]. Neurodevelopmental comorbidity was found in 28.5% of the MCDA group and 10% of the DCDA; however, this difference was not statistically significant in their study.

Chorionicity did not impact the risk of having a neurodevelopmental disorder in the surviving twins after birth in twin pregnancies with SIUFD in our study. It was found to occur in 8.3% of the MCDA group and 7.2% of the DCDA group. However, delayed developmental milestones were found more in MCDA co-twins (12.5%; 3/24) than in DCDA co-twins (5.8%; 4/69), but the difference was not statistically significant. Besides, the gestation age at SIUFD was not found to be associated with the occurrence of neurodevelopmental disorder in the surviving twin. Maternal deaths were seen in two pregnancies which was independent of the chorionicity of twin pregnancy. We could not find any significant association due to the small study population.

We found that twin pregnancies with SIUFD have an increased incidence of preterm labour, increased neonatal death of the surviving twin, and neurodevelopmental disorders. Monochorionicity and SIUFD at < 28 weeks of gestation are associated with increased neonatal deaths in such populations. Chorionicity was found to have no association with neurodevelopmental disorders in the surviving twin, but developmental delay was found more in the monochorionic group. To the best of our knowledge, this is the first study from the Indian population addressing the prognosis of the co-twin after SIUFD in twin pregnancies.

Our study had a few limitations. The study being retrospective could not have included the whole patient population for assessment of neonatal prognosis. Standardized care may not have been provided to all the neonatal population. Neuroimaging was not done in all the cases and hence not included in the data.

## Conclusions

Twin pregnancies are known to have an increased risk of maternal, fetal, and neonatal complications. Monochorionicity is associated with an increased risk of SIUFD and fetal morbidity. SIUFD in twin pregnancies is associated with co-twin death, prematurity, and neurological damage. We also found that twin pregnancies with SIUFD have an increased incidence of preterm labour, increased neonatal death of the surviving twin, and neurodevelopmental disorders. Monochorionicity and SIUFD at < 28 weeks of gestation were associated with increased neonatal deaths in such populations. Chorionicity had no association with neurodevelopmental disorders in the surviving twin; however, developmental delay was found more in the monochorionic group.

## References

[1] Pharoah PO, Adi Y (2000). Consequences of in-utero death in a twin pregnancy. Lancet.

[2] Hillman SC, Morris RK, Kilby MD (2011). Co-twin prognosis after single fetal death: a systematic review and meta-analysis. Obstet Gynecol.

[3] Cleary-Goldman J, D'Alton M (2004). Management of single fetal demise in a multiple gestation. Obstet Gynecol Surv.

[4] Saito K, Ohtsu Y, Amano K, Nishijima M (1999). Perinatal outcome and management of single fetal death in twin pregnancy: a case series and review. J Perinat Med.

[5] Mackie FL, Rigby A, Morris RK, Kilby MD (2019). Prognosis of the co-twin following spontaneous single intrauterine fetal death in twin pregnancies: a systematic review and meta-analysis. BJOG.

[6] Ong SS, Zamora J, Khan KS, Kilby MD (2006). Prognosis for the co-twin following single-twin death: a systematic review. BJOG.

[7] Shek NW, Hillman SC, Kilby MD (2014). Single-twin demise: pregnancy outcome. Best Pract Res Clin Obstet Gynaecol.

[8] Morris RK, Mackie F, Garces AT, Knight M, Kilby MD (2020). The incidence, maternal, fetal and neonatal consequences of single intrauterine fetal death in monochorionic twins: a prospective observational UKOSS study. PLoS One.

[9] Blickstein I, Perlman S (2013). Single fetal death in twin gestations. J Perinat Med.

[10] Malinowski W, Koktysz R, Stawerski P (2005). The case of monochorionic twin gestation complicated by intrauterine demise of one fetus in the first trimester. Twin Res Hum Genetics.

[11] Bajoria R, Wee LY, Anwar S, Ward S (1999). Outcome of twin pregnancies complicated by single intrauterine death in relation to vascular anatomy of the monochorionic placenta. Hum Reprod.

[12] Fusi L, Gordon H (1990). Twin pregnancy complicated by single intrauterine death. Problems and outcome with conservative management. Br J Obstet Gynaecol.

[13] de la Calle M, Bartha JL, Serrano H, Ramiro-Cortijo D (2021). Obstetric outcomes in the surviving fetus after intrauterine fetal death in bichorionic twin gestations. Children (Basel).

[14] D'Antonio F, Thilaganathan B, Dias T, Khalil A (2017). Influence of chorionicity and gestational age at single fetal loss on risk of preterm birth in twin pregnancy: analysis of STORK multiple pregnancy cohort. Ultrasound Obstet Gynecol.

[15] Fichera A, Zambolo C, Accorsi P, Martelli P, Ambrosi C, Frusca T (2009). Perinatal outcome and neurological follow up of the cotwins in twin pregnancies complicated by single intrauterine death. Eur J Obstet Gynecol Reprod Biol.

